# Asthma in Inner-City Children at 5–11 Years of Age and Prenatal Exposure to Phthalates: The Columbia Center for Children’s Environmental Health Cohort

**DOI:** 10.1289/ehp.1307670

**Published:** 2014-09-17

**Authors:** Robin M. Whyatt, Matthew S. Perzanowski, Allan C. Just, Andrew G. Rundle, Kathleen M. Donohue, Antonia M. Calafat, Lori A. Hoepner, Frederica P. Perera, Rachel L. Miller

**Affiliations:** 1 Department of Environmental Health Sciences, Columbia Center for Children’s Environmental Health, Mailman School of Public Health, Columbia University, New York, New York, USA; 2 Department of Environmental Health, Harvard School of Public Health, Boston, Massachusetts, USA; 3 Department of Epidemiology, Mailman School of Public Health, Columbia University, New York, New York, USA; 4 Division of Pulmonary, Allergy, and Critical Care, Department of Medicine, Columbia University College of Physicians and Surgeons, New York, New York, USA; 5 National Center for Environmental Health, Centers for Disease Control and Prevention, Atlanta, Georgia, USA; 6 Division of Allergy and Immunology, Department of Pediatrics, Columbia University, College of Physicians and Surgeons, New York, New York, USA

## Abstract

Background: Studies suggest that phthalate exposures may adversely affect child respiratory health.

Objectives: We evaluated associations between asthma diagnosed in children between 5 and 11 years of age and prenatal exposures to butylbenzyl phthalate (BBzP), di-*n*-butyl phthalate (DnBP), di(2-ethylhexyl) phthalate (DEHP), and diethyl phthalate (DEP).

Methods: Phthalate metabolites were measured in spot urine collected from 300 pregnant inner-city women. Children were examined by an allergist or pulmonologist based on the first parental report of wheeze, other respiratory symptoms, and/or use of asthma rescue/controller medication in the preceding 12 months on repeat follow-up questionnaires. Standardized diagnostic criteria were used to classify these children as either having or not having current asthma at the time of the physician examination. Children without any report of wheeze or the other asthma-like symptoms were classified as nonasthmatics at the time of the last negative questionnaire. Modified Poisson regression analyses were used to estimate relative risks (RR) controlling for specific gravity and potential confounders.

Results: Of 300 children, 154 (51%) were examined by a physician because of reports of wheeze, other asthma-like symptoms, and/or medication use; 94 were diagnosed with current asthma and 60 without current asthma. The remaining 146 children were classified as nonasthmatic. Compared with levels in nonasthmatics, prenatal metabolites of BBzP and DnBP were associated with a history of asthma-like symptoms (*p* < 0.05) and with the diagnosis of current asthma: RR = 1.17 (95% CI: 1.01, 1.35) and RR = 1.25 (95% CI: 1.04, 1.51) per natural log-unit increase, respectively. Risk of current asthma was > 70% higher among children with maternal prenatal BBzP and DnBP metabolite concentrations in the third versus the first tertile.

Conclusion: Prenatal exposure to BBzP and DnBP may increase the risk of asthma among inner-city children. However, because this is the first such finding, results require replication.

Citation: Whyatt RM, Perzanowski MS, Just AC, Rundle AG, Donohue KM, Calafat AM, Hoepner LA, Perera FP, Miller RL. 2014. Asthma in inner-city children at 5–11 years of age and prenatal exposure to phthalates: the Columbia Center for Children’s Environmental Health Cohort. Environ Health Perspect 122:1141–1146; http://dx.doi.org/10.1289/ehp.1307670

## Introduction

Phthalates are high-production chemicals widely used in consumer products (Sathyanarayana 2008). Exposures are ubiquitous, including among inner-city populations [Centers for Disease Control and Prevention (CDC) 2009; Whyatt et al. 2012]. Diet is thought to be the main source of exposure for di(2-ethylhexyl) phthalate (DEHP), although nondietary pathways also can be substantial for other phthalates (Carlstedt et al. 2013; Koch 2013). Phthalates have short biologic half-lives, with most metabolites eliminated within 24 hr (Wittassek and Angerer 2008). Measures of phthalate metabolites in the urine are informative as internal dosimeters of exposure because urinary enzymatic activity is negligible (Kato et al. 2004). Thus, metabolite concentrations in urine reflect an individual’s internal exposure to phthalates, rather than phthalate contaminants introduced into their urine sample during collection and processing. Prior studies have shown moderate intraclass correlation coefficients (ICCs) for most phthalate metabolites in repeat urine samples indicating reasonable reliability (Hauser et al. 2004; Teitelbaum et al. 2008; Whyatt et al. 2012).

Preliminary epidemiologic findings suggest that phthalates may be associated with child asthma and other respiratory problems (Bornehag and Nanberg 2010; Kwak et al. 2009). Among Swedish children 3–8 years of age, house dust concentrations of DEHP were associated with physician-confirmed asthma, and concentrations of butylbenzyl phthalate (BBzP) were associated with child eczema and rhinitis (Bornehag et al. 2004). A follow-up study from Bulgaria reported that house dust DEHP concentrations were associated with child asthma (Kolarik et al. 2008). In cross-sectional analyses, urinary concentrations of diethyl phthalate (DEP) and di-*n*-butyl phthalate (DnBP) metabolites were associated with decreased forced expiratory volume in 1 sec (FEV_1_) in adult males but not adult females (Hoppin et al. 2004). We previously reported a statistically significant association between fractional exhaled nitric oxide (FeNO), a measure of airway inflammation, and concentrations of metabolites of DEP and BBzP, in urine collected from cohort children at 5–9 years of age (Just et al. 2012a). In addition, two studies have shown that polyvinyl chloride (PVC) materials in the home, exposure sources for BBzP and DEHP, are associated with child asthma and other respiratory symptoms (Bornehag and Nanberg 2010; Larsson et al. 2010).

Increasingly, it is becoming evident that the prenatal period is an important window of susceptibility when environmental exposures may affect lung development and respiratory health (Duijts 2012; Miller et al. 2001; Rosa et al. 2011). However, to our knowledge no prior studies have been published on effects of prenatal phthalate exposures and child asthma. The current study was designed to fill this gap. We hypothesized that maternal prenatal urinary metabolite concentrations of BBzP, DnBP, DEHP, and DEP would be associated with current asthma among inner-city children.

## Methods

The study includes 300 inner-city women and their children, 5–11 years of age, participating in the Columbia Center for Children’s Environmental Health (CCCEH) longitudinal birth cohort of 727 women enrolled between 1998 and 2006 while they were pregnant with the index child. Women 18–35 years old, who self-identified as African American or Dominican and had resided in northern Manhattan or the South Bronx for at least 1 year before pregnancy, were enrolled through prenatal clinics at Harlem and New York Presbyterian Hospital (Perera et al. 2003). Women were excluded from enrollment into the cohort if they reported active smoking, used other tobacco products or illicit drugs, had diabetes, hypertension, or known HIV, or had their first prenatal visit after 20 weeks gestation. The 300 children were included in the present analysis if phthalate metabolite concentrations had been measured in a maternal spot urine sample collected during pregnancy and data were available for model covariates and to classify the child’s asthma status. Children were excluded from the present analysis if their mothers reported active smoking during pregnancy (*n* = 30), if prenatal maternal urine phthalate metabolite concentrations were not available (*n* = 281), if children were lost to follow-up (*n* = 89), or were missing covariate data (*n* = 27). The 300 children were similar to the other children in the CCCEH cohort with regard to their race/ethnicity, maternal prenatal marital status and education level, household income, household tobacco smoke exposure, maternal asthma, or maternal demoralization during pregnancy (see Supplemental Material, Table S1). All women signed an IRB-approved consent form and children signed an IRB-approved assent form beginning at age 7. The institutional review boards at the Columbia University Medical Center and the CDC approved the study.

*Urine sample collection and phthalate measurements*. A spot urine sample was collected from the women (*n* = 300) during the third trimester (mean ± SD, 34.0 ± 3.0 weeks gestation; median, 33.7 weeks) and from the children at ages 3 (*n* = 216), 5 (*n* = 270) and 7 years (*n* = 154). Samples were analyzed for the following four phthalate metabolites at the CDC using solid phase extraction coupled with high performance liquid chromatography–isotope dilution tandem spectrometry as described (Kato et al. 2005): mono(2-ethyl-5-hydroxyhexyl) phthalate (MEHHP), as a representative of the DEHP metabolites as described (Just et al. 2012a); monobenzyl phthalate (MBzP), a metabolite of BBzP; mono-*n*-butyl phthalate (MnBP), a metabolite of DnBP; and monoethyl phthalate (MEP), a metabolite of DEP. Bisphenol A (BPA) was measured in maternal and child urine samples using solid phase extraction coupled with high performance liquid chromatography-isotope tandem mass spectrometry (Ye et al. 2005). Specific gravity was measured in the urine samples using a handheld refractometer and was used to control for urinary dilution (Atago PAL 10-S; Bellevue, WA) (Hauser et al. 2004).

*Diagnosis of child asthma*. At child ages 5, 6, 7, 9, and 11 years, repeat questionnaires were administered to the mother or guardian to gather information on asthma-like symptoms in the child. The questionnaires were administered in English or Spanish by fully bilingual research workers. These included the well-validated International Study of Asthma and Allergies in Childhood (ISAAC) questionnaire (Asher et al. 1995) and the Brief Respiratory Questionnaire (BRQ) (Bonner et al. 2006), as well as report of asthma rescue and/or controller medication. As described previously (Donohue et al. 2013), children were referred for physician evaluation for asthma based on the first report that the child had wheeze or whistling in the chest, a cough that lasted more than a week, other breathing problems, and/or use of asthma rescue or controller medication in the preceding 12 months on any of the follow-up study questionnaires. Examinations included a standardized history, physical examination, and prebronchodilator and postbronchodilator pulmonary function testing. Two allergists/pulmonologists independently reviewed the results of each examination and classified their asthma status according to the following pre-specified criteria: *a*) current asthma symptoms OR current asthma medication AND either a 12% increase in FEV_1_ or a 30% increase in FEF_25–75_ postbronchodilator; *b*) current asthma symptoms OR current asthma medication AND history of asthma symptoms on previous questionnaires; *c*) history of asthma symptoms on previous questionnaires AND wheeze on current exam.

The physician examination was conducted once during study follow-up, and the children were classified as having current asthma or as not having current asthma based on their status at the time of the examination. Children whose parents or guardians did not report wheeze or the other asthma-like symptoms or asthma medication use on any of the follow-up questionnaires were classified as nonasthmatics at the time of the last negative questionnaire.

*Statistical analyses*. Phthalate metabolite concentrations were right-skewed and transformed using the natural logarithm (ln). Concentrations that were below the limit of detection (LOD) were assigned a value of 0.5 ×⊇LOD. Variables assessed as potential confounders were selected from those known or suspected of being associated with phthalate concentrations or asthma (Just et al. 2012a; Miller et al. 2004; Whyatt et al. 2012). Variables were retained in the models if they were associated with the outcome (*p* ≤ 0.10) and/or addition or removal changed the coefficient for the exposure (phthalate metabolite) to outcome relationship by > 10%. Variables assessed included race/ethnicity, prenatal and postnatal household tobacco smoke exposure, maternal history of asthma, maternal education, maternal marital status, material hardship (lack of food, housing, gas, or electricity, clothing, or medicine during pregnancy), sex of the child, and child age at diagnosis (age at diagnosis as either current asthma or not current asthma for children with a history of the asthma-like symptoms seen by the physician, or age at classification as nonasthmatic at the last negative questionnaire for children without history of the asthma-like symptoms). Maternal prenatal demoralization also was assessed by validated questionnaire (Dohrenwend et al. 1978) because it previously has been associated with wheeze among children in the cohort (Reyes et al. 2011). Models were also adjusted for maternal prenatal BPA urinary concentrations (Donohue et al. 2013) but not child postnatal BPA concentrations because inclusion caused < 10% change in the phthalate exposure–outcome relationship. In addition, we assessed whether child postnatal urinary phthalate concentrations acted as a confounder using the phthalates measured at child ages 3, 5, and 7 years (2 children who had phthalates measured in urine collected at age 7 but whose asthma status was determined before age 7 were removed from the age 7 analyses). However, none of the postnatal phthalate metabolite concentrations were associated with child asthma (see Supplemental Material, Table S2), and inclusion caused < 10% change in effect size of predictor variables. The maternal phthalate urinary concentrations were included in models as ln-transformed variables, and also were categorized into tertiles and modeled using indicator variables to estimate relative risks comparing the second and third tertiles to the first tertile (referent). Metabolite concentrations were adjusted for specific gravity before categorizing using the formula described previously (Hauser et al. 2004). Consistent with our prior approach (Just et al. 2012a), relative risks were estimated using Poisson regression with robust standard error estimation using the generalized estimating equations based method of Zou (2004) (see also Lovasi et al. 2012; Spiegelman and Hertzmark 2005). Analyses were conducted using SPSS version 21 (SPSS, IBM, Chicago, IL). Results were considered significant at *p* < 0.05.

## Results

Table 1 provides subject characteristics. Self-reported ethnicity was African American (35.7%) or Domincan (64.3%). Educational attainment was low (35.7% had not completed high school), and the majority (68%) reported never having been married. Table 2 shows the distribution of the urinary phthalate metabolite concentrations measured in maternal prenatal spot urine. All phthalate metabolites were detected in 100% of maternal prenatal urine samples except for MBzP in one sample (assigned a value of 0.11 ng/mL). Concentrations were generally comparable with those of a representative sample of the U.S. population sampled over roughly the same time period (1999–2004) (CDC 2009). Table 3 shows the correlations between the (ln)maternal prenatal phthalate metabolite concentrations adjusted for specific gravity. All metabolites were positively correlated, with correlation coefficients ranging from 0.16 (for MEP and MnBP) to 0.50 (for MnBP and MBzP) (all *p*-values < 0.01).

**Table 1:** Characteristics of children (*n* = 300) from the CCCEH birth cohort.

Characteristic	*n* (%) or mean ± SD
Maternal age (years)	25.3 ± 4.8
Maternal asthma history	76 (25.3)
Maternal demoralization^*a*^	1.1 ± 0.65
Ethnicity	
African American	107 (35.7)
Dominican	193 (64.3)
Maternal education	
< High school	107 (35.7)
High school or general educational development	114 (38.0)
> High school	79 (26.3)
Marital status	
Never married	204 (68)
Married^*b*^	81 (27.0)
Separated, widowed, divorced	15 (5.0)
Household smoke exposure^*c*^	154 (51.3)
Prenatal urinary bisphenol A (ng/mL)	3.1 ± 4.3
Child age at assessment (years)	8.1 ± 1.9
Child sex (% female)	163 (54.3)
^***a***^Mean of 27 items each on 5-point Likert scale (0–4) (Dohrenwend et al. 1978). ^***b***^Includes women living with the same partner for > 7 years. ^***c***^Whether or not others smoked in the home during the prenatal period and whether or not the mother and/or others smoked in the home during childhood gathered by repeat questionnaire.	

**Table 2:** Distribution of phthalate metabolites in maternal prenatal urine during pregnancy (ng/mL).

	Geometric mean (95% CI)	Concentration (ng/mL)		
		25%	50%	75%
MEHHP	22.4 (19.4, 25.9)	10.6	21.6	50.0
MBzP^*a*^	13.5 (11.6, 15.6)	5.8	15.0	31.9
MnBP	37.5 (33.2, 42.3)	19.3	38.3	80.5
MEP	160.3 (139.6, 184.0)	68.0	143.5	335.2
^***a***^All metabolites were above the LOD except for one sample for MBzP, which was imputed (LOD × 0.05).				

**Table 3:** Correlation^*a*^ between prenatal phthalate metabolite concentrations adjusted for specific gravity (*n* = 300).

	(ln)MnBP	(ln)MEP	(ln)MEHHP
(ln)MBzP	0.50*	0.16*	0.27*
(ln)MnBP		0.27*	0.30*
(ln)MEP			0.17*
^***a***^Spearman’s rank. *p < 0.01.			

A total of 1,013 repeat questionnaires on wheeze and other asthma-like symptoms were administered between ages 5 and 11 years (mean, 3.7 ± 1.1 questionnaires per child). The children were 8.1 ± 1.9 years on average at the time of the last questionnaire administration. Of the 300 children, 154 had a history of the following asthma-like symptoms on one or more questionnaires between 5 and 11 years: a report in the preceding 12 months of wheeze or whistling in the chest, a cough that lasted more than a week, other breathing problems, and/or reported use of asthma rescue or controller medication. These 154 children met our screening criteria for the physician diagnosis of asthma based on the first report of any of these symptoms. At the time of the physician exam, 94 children were diagnosed with current asthma and 60 were diagnosed as not having current asthma. A greater proportion of African-American than Dominican children were classified as having current asthma (36% vs. 29%, chi-square *p* = 0.11). The remaining 146 children had no history of any of the asthma-like symptoms on repeat questionnaires and were classified as nonasthmatic at the time of the last negative questionnaire.

**Figure 1:**
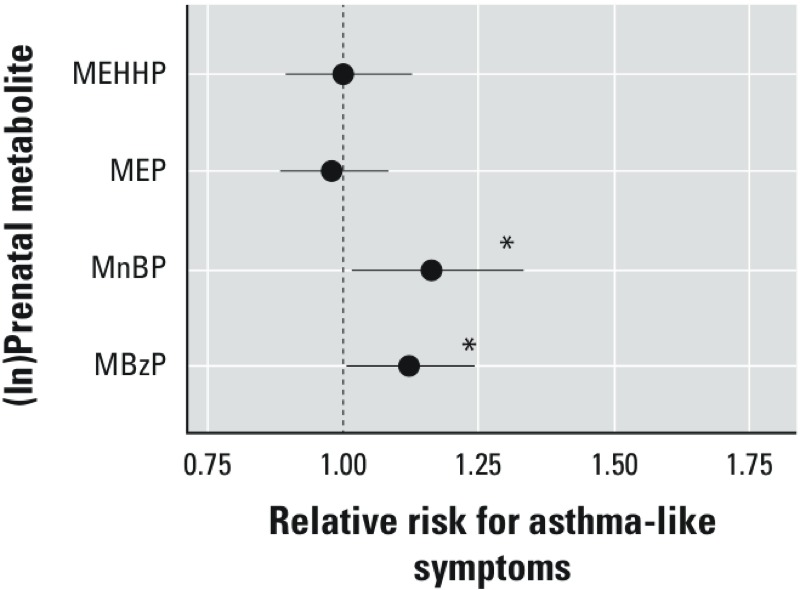
Association between maternal prenatal (ln)phthalate metabolite concentrations and presence (*n *= 154) compared with the absence (*n *= 146) of a history of asthma-like symptoms on repeat questionnaires administered between child ages 5 and 11 years. RRs were estimated using Poisson regression with robust standard error estimation using the generalized estimating equations controlling for maternal asthma, household tobacco smoke exposure, maternal prenatal BPA, maternal prenatal demoralization, and maternal prenatal specific gravity. *p < 0.05.

**Figure 2:**
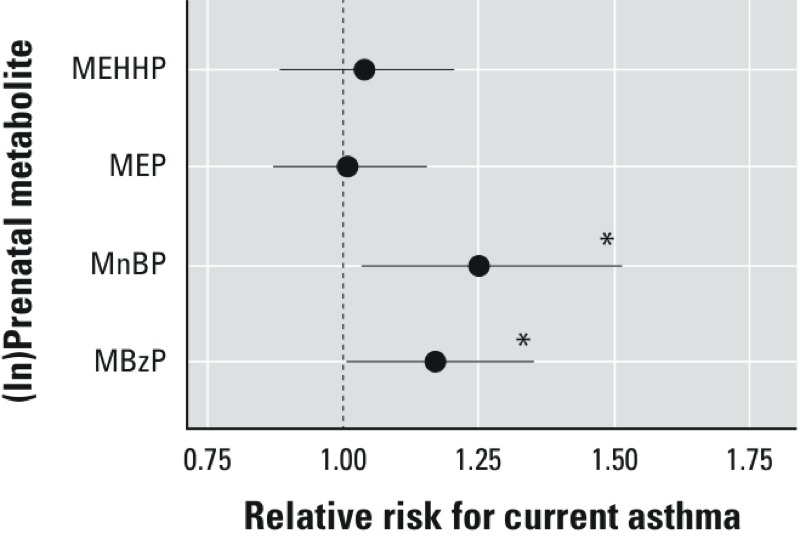
Association between maternal prenatal (ln)phthalate metabolite concentrations and diagnosis of current asthma (*n *= 94) compared with nonasthmatic (*n *= 146) between child ages 5 and 11 years. RRs were estimated using Poisson regression with robust standard error estimation using the generalized estimating equations controlling for maternal asthma, household tobacco smoke exposure, maternal prenatal BPA, maternal prenatal demoralization, child age, and maternal prenatal specific gravity. *p < 0.05.

**Figure 3:**
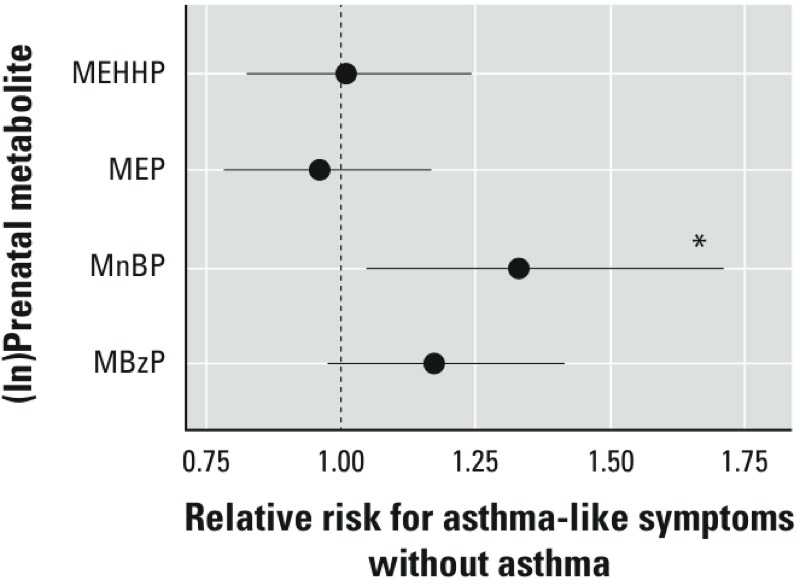
Association between maternal prenatal (ln)phthalate metabolites concentrations and diagnosis as not current asthma among children with a history of the asthma-like symptoms (*n *= 60) compared with children without any history of asthma-like symptoms classified as nonasthmatics (*n *= 146). RRs were estimated using Poisson regression with robust standard error estimation using the generalized estimating equations controlling for maternal asthma, household tobacco smoke exposure, maternal prenatal BPA, maternal prenatal demoralization, child age, and maternal prenatal specific gravity. *p < 0.05.

We estimated relative risks (RRs) for three different outcome groups relative to the children classified as nonasthmatic (*n* = 146) as a common referent group: *a*) children with any report of symptoms and/or medication use, regardless of the outcome of their asthma examination (*n* = 154); *b*) children diagnosed with current asthma at the time of their physician examination (*n* = 94); and *c*) children with a history of symptoms but diagnosed as not having current asthma at the time of the physician examination (*n* = 60). Linear results are presented in Figures 1–3 and categorical results in Table 4.

**Table 4:** RR (95% CI) for history of asthma-like symptoms (group 1, *n *= 154), diagnosis of current asthma (group 2, *n *= 94), and diagnosis of not current asthma (group 3, *n *= 60) compared with nonasthmatics (*n *= 146) by tertiles of phthalate metabolites.

Outcome group and exposure	MBzP		MnBP		MEP		MEHHP	
	Case/control (*n*)	RR (95% CI)	Case/control (*n*)	RR (95% CI)	Case/control (*n*)	RR (95% CI)	Case/control (*n*)	RR (95% CI)
Group 1
1st tertile	44/57	Referent	46/60	Referent	46/52	Referent	57/47	Referent
2nd tertile	55/47	1.25 (0.94, 1.65)	53/41	1.39 (1.06, 1.82)*	59/42	1.33 (1.03, 1.73)*	46/47	0.92 (0.71, 1.20)
3rd tertile	55/42	1.39 (1.05, 1.86)*	55/45	1.44 (1.09, 1.90)*	49/52	1.08 (0.82, 1.42)	51/52	0.97 (0.74, 1.28)
Group 2
1st tertile	25/57	Referent	27/60	Referent	29/52	Referent	34/47	Referent
2nd tertile	34/47	1.31 (0.87, 1.98)	38/41	1.87 (1.28, 2.67)**	35/42	1.11 (0.97, 1.27)	27/47	0.98 (0.85, 1.12)
3rd tertile	35/42	1.72 (1.15, 2.59)**	29/45	1.78 (1.18, 2.70)**	30/52	1.04 (0.91, 1.19)	33/52	1.03 (0.89, 1.20)
Group 3
1st tertile	19/57	Referent	19/60	Referent	17/52	Referent	23/47	Referent
2nd tertile	21/47	1.34 (0.80, 2.25)	15/41	1.46 (0.81, 2.65)	24/42	1.15 (0.99, 1.34)	19/47	0.98 (0.84, 1.13)
3rd tertile	20/42	1.44 (0.83, 2.49)	26/45	1.71 (1.02, 2.88)*	19/52	1.04 (0.91, 1.89)	18/52	0.95 (0.82, 1.10)
Models compare children in each outcome group with children without history of asthma-like symptoms controlling for maternal asthma, household smoke exposure, maternal prenatal BPA, maternal prenatal demoralization, maternal prenatal specific gravity, and child age (for outcome groups 2 and 3). *p < 0.05. **< 0.01.								

*Report of asthma-like symptoms regardless of the outcome of the asthma examination*. Compared with nonasthmatic children, a significant association was seen between maternal prenatal urinary MBzP and MnBP concentrations and children with a history of asthma-like symptoms: RR = 1.12 (95% CI: 1.01, 1.24) and RR = 1.16 (95% CI: 1.02, 1.33) per ln-unit increase, respectively (Figure 1). Compared with children of mothers with MBzP and MnBP concentrations in the first tertile, the RRs for a history of asthma-like symptoms among those in the second tertile were 1.25 (95% CI: 0.94, 1.65) and 1.39 (95% CI: 1.06, 1.82), respectively, and among those in the third tertile the RRs were 1.39 (95% CI: 1.05, 1.86) and 1.44 (95% CI: 1.09, 1.90, respectively (Table 4). There was no significant increase in risk of asthma-like symptoms in the linear models associated with either maternal MEP or MEHHP concentrations (Figure 1). However, in the categorical models (Table 4) compared with children of mothers with MEP in the first tertile, a significant increase in risk of asthma-like symptoms was seen among those in the second tertile (RR = 1.33; 95% CI: 1.03, 1.73) but not among those in the third tertile (RR = 1.08; 95% CI: 0.82, 1.42). There was no increase in risk of asthma-like symptoms across tertiles of MEHHP concentrations (Table 4).

*Diagnosis of current asthma at physician examination*. Compared with nonasthmatic children, maternal prenatal MBzP and MnBP concentrations, but not the other phthalate metabolites, were associated with the diagnosis of the child with current asthma at the physician examination in the linear models: RR = 1.17 (95% CI: 1.01, 1.35) and RR = 1.25 (95% CI: 1.04, 1.51) per ln-unit increase, respectively (Figure 2). In the categorical models (Table 4), compared with children of mothers with MBzP and MnBP concentrations in the first tertile, RRs for current asthma among those in the second tertile were 1.31 (95% CI: 0.87, 1.96) and 1.87 (95% CI: 1.28, 2.67), respectively, and among those in the third tertile RRs were 1.72 (95% CI: 1.15, 2.59) and 1.78 (95% CI: 1.18, 2.70), respectively. There were no significant associations between diagnosis of current asthma and maternal prenatal MEP or MEHHP concentrations in either linear (Figure 2) or categorical (Table 4) models.

*History of symptoms but diagnosis of not current asthma at physician examination*. Compared with nonasthmatic children, maternal prenatal MnBP concentrations were associated with a history of asthma-like symptoms but diagnosis of not current asthma at the physician examination: RR = 1.33 (95% CI: 1.05, 1.70) per ln-unit increase (Figure 3). In the categorical models (Table 4), compared with mothers with MnBP concentrations in the first tertile, the RR for not current asthma for those in the second tertile was 1.46 (95% CI: 0.81, 2.65) and in the third tertile was 1.71 (1.02, 2.88). The association between maternal prenatal MBzP concentrations and diagnosis of not current asthma among those with a history of the asthma-like symptoms was of borderline significance in the linear model: RR 1.17 (95% CI: 0.98, 1.41) per ln-unit increase (Figure 3). However, there was no increase in risk across tertiles of maternal MBzP concentration, and there were no significant associations between diagnosis of not current asthma and maternal prenatal MEP or MEHHP concentrations in either linear (Figure 3) or categorical (Table 4) models.

## Discussion

We observed a significant association between concentrations of MBzP, the BBzP metabolite, and MnBP, the DnBP metabolite, in maternal urine collected during the third trimester of pregnancy and diagnosis of current asthma among CCCEH cohort children 5–11 years of age. Risk of current asthma was > 70% higher among children whose mothers had BBzP and DnBP metabolite concentrations during pregnancy in the third versus first tertile. This work extends our previous CCCEH findings in cross-sectional analyses that increases in concentrations of metabolites of BBzP, as well as DEP, in child urine were associated with increases in FeNO, an indicator of airway inflammation and asthma (Just et al. 2012a). Although prior research has shown associations between other phthalates and child asthma, only one prior cross-sectional study reported evidence of a dose–response relationship between MBzP and child asthma but was limited by small sample size (*n* = 101) (Hsu et al. 2012). Other cross-sectional studies in Sweden (*n* = 400 children ages 3–8 years) and Bulgaria (*n* = 102 children ages 2–7 years) have reported associations between house dust concentrations of DEHP and child asthma, and of BBzP and child eczema and rhinitis, but not BBzP and child asthma (Bornehag et al. 2004; Kolarik et al. 2008). A recent cross-sectional study reported a significant association between child asthma and mono(carboxyoctyl) phthalate and mono(carboxynonyl) phthalate in first morning void urine collected from 623 10-year-old Norwegian children, but did not observe any association between asthma and urinary concentrations of the BBzP or DnBP metabolites (Bertelsen et al. 2013). Another recent cross-sectional study conducted among participants in NHANES (National Health and Nutrition Examination Study) found urinary concentrations of MBzP, but not MnBP, to be significantly associated with current asthma, wheeze, hay fever, and rhinitis in adults (*n* = 1,546) but not in children 6–17 years of age (*n* = 779) (Hoppin et al. 2013).

Consistent with our prior findings (Whyatt et al. 2012), significant correlations were seen between prenatal urinary concentrations of MBzP and MnBP adjusted for specific gravity (Spearman’s rho = 0.50). This may reflect common sources of exposure and/or similar metabolic pathways. Consumer products including personal care products and home materials are sources of exposure to both of these phthalates (Buckley et al. 2012; Carlstedt et al. 2013). In addition, MnBP is a minor metabolite of BBzP, accounting for approximately 6% (Anderson et al. 2001). Nonetheless, most MnBP in urine is attributable to DnBP exposures. Several studies, including findings in the CCCEH cohort, suggest that PVC materials in the home are likely a substantial source of BBzP exposure, as indicated by measures in maternal and child urine and residential and personal air samples (Adibi et al. 2008; Carlstedt et al. 2013). Although DEHP is also a constituent of PVC, evidence suggests that exposure to this phthalate occurs primarily through the diet, likely as a result of use in food packaging (Koch 2013; Rudel et al. 2011). Prior cross-sectional studies also have found PVC materials in the home to be associated with child asthma and other respiratory symptoms (Bornehag and Nanberg 2010), and these results provide at least some corroboration for the current findings. For example, in a prospective study of 4,779 children in Sweden without asthma or respiratory symptoms at child ages 1–3 years, PVC flooring at baseline was associated with parental report of child asthma at the 5- and 10-year follow-up (Larsson et al. 2010; Shu et al. 2014).

The significant association seen in our present study between MnBP and MBzP urinary concentrations and asthma-like symptoms, regardless of whether or not the child received a diagnosis of current asthma, was not anticipated. These findings may imply that prenatal exposure to some phthalates has effects on transient wheeze and/or nonspecific airway hyperresponsiveness. It is possible that the respiratory consequences of prenatal exposure to phthalates mimic what has been observed following prenatal exposure to cigarette smoke, where several large cohort studies have essentially established its role in recurrent wheeze in very young children (Magnusson et al. 2005). Alternatively, prenatal phthalate exposure may induce a nonspecific airway hyperresponsiveness, manifested as report of wheeze, use of asthma medication, cough, or other breathing problems, that develops into clinical asthma during childhood only in a subset of children. The development of airway hyperresponsiveness is believed to have an environmental component (Lund et al. 2007; Riley et al. 2012), and develops at a very early age (Lesouef et al. 1989). Further prospective studies are needed to resolve these important clinical questions.

Research on mechanisms whereby phthalates might induce asthma or asthma-like symptoms is extremely limited [reviewed by (Kwak et al. 2009)]. Several phthalates have shown adjuvant effects on proallergic T helper 2 differentiation and immunoglobulin (Ig) G_1_ and IgE production when administered via subcutaneous or intraperitoneal injection to BALB/c mice sensitized by ovalbumin (Bornehag and Nanberg 2010; Guo et al. 2012). Our prior research in the CCCEH cohort did not find any association between maternal prenatal urinary MBzP concentrations and child seroatopy, although we did observe an association between maternal prenatal MBzP concentrations and early-onset eczema (Just et al. 2012b). In addition, as discussed above, when we used a repeated-measures design, we saw a significant association between MBzP concentrations in child urine and FeNO, suggesting that MBzP induces airway inflammation (Just et al. 2012a). In cross-sectional analyses, urinary concentrations of MBzP also have been shown to be associated with C-reactive protein, a nonspecific marker of systemic inflammation, and both MBzP and MnBP have been associated with biomarkers of oxidative stress (Ferguson et al. 2011, 2012). However, the relevance of these findings to the potential effect of the phthalates on respiratory health remains unclear. Mechanistic data explaining why the prenatal period of exposure may be deleterious also are limited, but our group has provided evidence of epigenetic regulation following prenatal exposure to several environmental exposures in human and mouse studies on asthma (Kundakovic 2013; Liu et al. 2008; Niedzwiecki et al. 2012; Perera et al. 2009; Tang et al. 2012).

Strengths of the current study include the standardized physician diagnosis of child asthma that is an improvement over most prior research, which used parental report of child asthma as the outcome. Additionally, this is the first study to evaluate associations between prenatal phthalate exposure and asthma during early to mid-childhood in a longitudinal birth cohort. This is a strength because most prior studies of phthalates and asthma have been cross-sectional. The rate of both maternal and child asthma in our cohort is high (31.8% and 25.2%, respectively). Asthma prevalence among New York City children ranges from 3% to 25% (Nicholas et al. 2005), with some inner-city communities having triple the asthma prevalence of their bordering neighborhoods. Although family history of asthma or atopy were not required inclusion criteria for the current cohort, pregnant mothers were informed at enrollment that the research was evaluating environmental risk factors in child asthma development, and this may have been an incentive for mothers whose children were at higher risk to participate.

Limitations also should be noted. We used as our exposure dosimeters the measurements of the phthalate metabolite concentrations in a single prenatal spot urine sample, and this could result in exposure misclassification, especially for some phthalate metabolites. Controlling for urinary dilution, we previously reported that the ICCs for phthalate metabolites in repeat prenatal spot urine samples (*n* = 135) collected biweekly over the last 6 weeks of pregnancy from 48 women in the CCCEH cohort were 0.77 for MBzP, 0.64 for MnBP, 0.27 for MEHHP, and 0.19 for MEP (Whyatt et al. 2012), indicating that reliability of the phthalate biomarkers differs across the phthalates. We would expect the exposure misclassification for these phthalates to be nondifferential with respect to asthma and could thus reduce our power to observe an effect for either MEP or MEHHP on asthma risk, because they had the lower ICCs. However, this is less likely to be a problem with MBzP and MnBP due to the higher ICCs in repeat prenatal urine samples, coupled with the relatively high correlations seen previously in the CCCEH cohort between BBzP concentrations in maternal 48-hr prenatal personal air samples and 2-week integrated indoor air samples (Spearman’s rho = 0.67) and between BBzP in maternal personal and indoor air samples and MBzP concentrations in maternal prenatal urine (Spearman’s rho = 0.48 and 0.71, respectively) (Adibi et al. 2008). However, the correlations between DnBP concentration in maternal prenatal personal and indoor air samples and MnBP concentrations in maternal prenatal urine were considerably lower (Spearman’s rho = 0.05 and 0.27, respectively) (Adibi et al. 2008). Missing data and loss to follow-up, as often occurs in a long-term prospective study, could also bias study results (Kurukulaaratchy et al. 2003; Matricardi et al. 2008). However, 97% of the children in the current study had questionnaire data on ISAAC wheeze and other respiratory symptoms collected at least twice, and 86% of the children had questionnaire data collected three or more times. In addition, maternal urine samples were collected during the third trimester of pregnancy, and this is another potential limitation given that the critical window of susceptibility is not known but may well be earlier in pregnancy. Additionally, the research was conducted in an inner-city cohort, with high rates of maternal and child asthma and was restricted to nonsmoking mothers during pregnancy. Therefore, results may well not be generalizable to other populations. Further, noncausal associations (e.g., due to confounding by some other factor associated with phthalates and asthma) cannot be ruled out. For all of these reasons, the findings should be interpreted with caution before replication in other cohorts that include evaluation of associations between child asthma and exposures during other trimesters of pregnancy.

## Conclusion

These results suggest that prenatal exposure to BBzP and DnBP may increase risk of childhood asthma. The findings raise new concerns that the presence of relatively ubiquitous environmental exposures may have deleterious respiratory effects. However, because, to our knowledge, this is the first study to evaluate associations between prenatal phthalate exposures and child asthma risk, results require replication.

## Supplemental Material

(138 KB) PDF
