# Supplementary material for: Asthma in Inner-City Children at 5–11 Years of Age and Prenatal Exposure to Phthalates: The Columbia Center for Children’s Environmental Health Cohort
Source: Environ Health Perspect. 2014 Sep 17;122(10):1141–6. doi: 10.1289/ehp.1307670 (PMC4181924; doi:10.1289/ehp.1307670)
Supplement: (138 KB) PDF [file ehp.1307670.s001.508.pdf]

Environmental Health Perspectives doi:10.1289/ehp.1307670

Supplemental Material

Asthma in Inner-City Children at 5-11 Years of Age and Prenatal Exposure to Phthalates: The Columbia Center for Children's Environmental Health Cohort

Robin M. Whyatt, Matthew S. Perzanowski, Allan C. Just, Andrew G. Rundle, Kathleen M. Donohue, Antonia M. Calafat, Lori A. Hoepner, Frederica P. Perera, and Rachel L. Miller

Table S1. Characteristics of study subjects from the Columbia Center for Children's Environmental Health longitudinal birth cohort included and excluded from the current analyses.

Characteristics	Included (n=300)	Excluded (n=397)	P-value
**Maternal age (years)**[a]	25.3 ± 4.8	25.0 ± 5.0 (n=397)	P=0.32[d]
**Maternal asthma history**	76 (25.3%)	67 (20.2%)	P=0.12[e]
**Missing**	0	65	
**Maternal demoralization**[a,b]	1.1 ± 0.65 (n=300)	1.2 ± 0.6 (n=353)	P=0.13[d]
**Missing**	0	24	
**Ethnicity**			
African American	107 (35.7%)	128 (32.2%)	P=0.34[e]
Dominican	193 (64.3%)	269 (67.8%)	
**Maternal education**			
< High school	107 (35.7%)	134 (34.9%)	P=0.83[e]
≥ High school	193 (64.3%)	250 (65.1%)	
**Missing**	0	13	
**Marital status**			
Never married	204 (68.0%)	248 (63.4%)	P=0.21[e]
Ever married[c]	96 (32.0%)	143 (36.6%)	
**Missing**	0	6	
**Household income**			
< 10,000	122 (44.2%)	165 (43.9%)	P=0.85[e]
≥ 10,000	154 (55.8%)	202 (55.0)	
**Missing**	24	21	
**Sex of the child (% female)**	163/300 (54.3%)	196/397 (49.4%)	P=0.19[e]

[a]Mean ± standard deviation. [b]Mean of 27 items each on 5 point Likert scale (0-4) (Dohrenwend et al. 1978). [c]Includes women living with the same partner for greater than 7 years. [d]Group T-test. [e]Chi-square.

Table S2. Associations [relative risk (95% confidence intervals)] between (ln)postnatal phthalate metabolite concentrations and children diagnosed with current asthma compared to non-asthmatics.

Metabolite	Child age 3 (n=73 cases, 99 controls)	Child age 5 (n=81cases, 112 controls)	Child age 7 (n=59 cases, 57 controls)
MEHHP	1.02 (0.85, 1.22)	1.07 (0.92, 1.24)	1.06 (0.91, 1.25)
MEP	1.10 (0.95, 1.26)	1.00 (0.88, 1.13)	0.90 (0.77, 1.05)
MnBP	1.01 (0.82, 1.25)	0.97 (0.83, 1.15)	0.97 (0.81, 1.17)
MBzP	0.97 (0.86, 1.11)	0.98 (0.86, 1.11)	0.96 (0.84, 1.10)

Relative risks were estimated using Poisson regression with robust standard error estimation using the generalized estimating equations controlling for maternal asthma, maternal prenatal demoralization, household tobacco smoke exposures, maternal urinary BPA, maternal urinary specific gravity, child age and child urinary specific gravity at each age point
